# Is acute kidney injury in the early phase of sepsis a sign of metabolic downregulation in tubular epithelial cells?

**DOI:** 10.1186/cc14366

**Published:** 2015-03-16

**Authors:** K Jin, H Li, J Volpe, D Emlet, N Pastor-Soler, MR Pinsky, BS Zuckerbraun, K Hallows, JA Kellum, H Gomez

**Affiliations:** 1 University of Pittsburgh, PA, USA

## Introduction

This study tested the hypothesis that the cellular response in the kidney to sepsis is characterized by early activation of AMP activated protein kinase (AMPK), and that such activation is temporally associated with downregulation of the epithelial sodium channel (B-ENaC).

## Methods

Fifteen C57BL/6 wildtype (WT) mice were subjected to cecal ligation and puncture (CLP), and sacrificed at 2, 6, 18, 24, or 48 hours. In addition, we pretreated three WT and three AMPK Beta 1 knockout mice with the AMPK activator AICAR (100 mg/kg intraperitoneal, 24 hours before CLP), and sacrificed 24 hours after CLP. Blood and tissue samples were collected for all animals. AMPK activation (pThr172), B-ENaC, and mitophagy (LC3 II/I) were examined by western blot of kidney lysates. Plasma creatinine (Scr) was assessed using ELISA.

## Results

**Figure 1:**
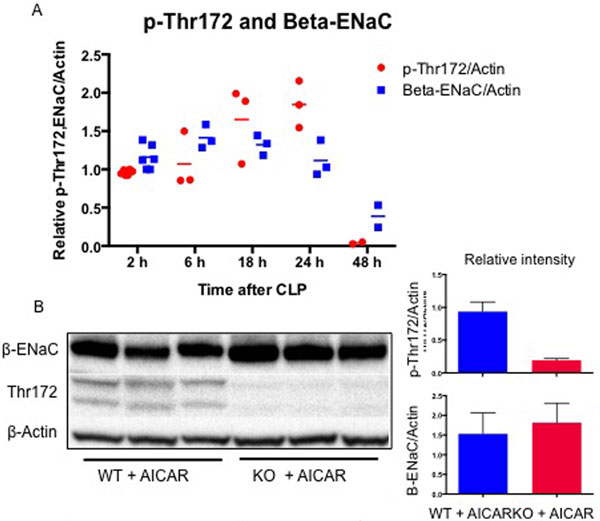
****(A) **AMPK activation (p-Thr172) and Beta-ENAC expression during early CLP**. **(B) **Difference in Beta-ENAC expression between AICAR pretreated WT versus KO at 24 hours after CLP.

The acute response to sepsis was characterized by early activation of AMPK which increased from 6 to 18 hours, peaked at 24 hours, and decreased by 48 hours (Figure 1A). This activation was associated with a consistent decrease in B-ENaC expression. In AICAR pretreated animals, AMPK was only activated in WT mice, which was associated with a decrease in the expression of B-ENaC as compared with AMPK KO mice (Figure 1B).

## Conclusion

AMPK was activated early after induction of sepsis, and was associated with a consistent decrease in Beta-ENaC expression in the apical membrane of tubular epithelial cells. In addition, absence of AMPK activation in KO animals was associated with increased expression of Beta-ENaC at 24 hours after CLP. These data support the hypothesis that early activation of AMPK decreases energy consumption through ion channel downregulation.

